# Hereditary angioedema

**DOI:** 10.1186/s12245-021-00364-7

**Published:** 2021-07-29

**Authors:** Helen Lesser, Jason E. Cohn

**Affiliations:** 1 Department of Otolaryngology-Head and Neck Surgery, Philadelphia College of Osteopathic Medicine, 4190 City Line Avenue, Philadelphia, PA 19131 USA; 2 Department of Otolaryngology-Head and Neck Surgery, Division of Facial Plastic Reconstructive Surgery, Louisiana State University Health Sciences Center, 1501 Kings Highway, Shreveport, LA 71103 USA

**Keywords:** Angioedema, Hereditary angioedema, Lip swelling, Pediatric otolaryngology

## Abstract

A 14-year-old African American female presented to the emergency department with spontaneous, sudden-onset lip swelling for 1 h. On examination, there was significant water-bag edema of the upper lip extending to the philtrum and premaxilla. Nasopharyngeal laryngoscopy revealed a patent airway without edema. She was initiated on intravenous dexamethasone, famotidine, and diphenhydramine, after which her edema improved but did not resolve. She was subsequently transferred to a local pediatric hospital and upon further testing she was found to have a C1 esterase inhibitor de novo gene mutation. Angioedema causes localized, non-pitting edema of the dermis, subcutaneous and submucosal tissue, and often manifests in the lips, face, mouth, and throat. Signs of laryngeal involvement include change in voice, stridor, dysphagia, and dyspnea. When laryngeal edema is present, it may necessitate definitive airway management and patients should be monitored in the intensive care unit.

## Case presentation

**Fig. 1:**
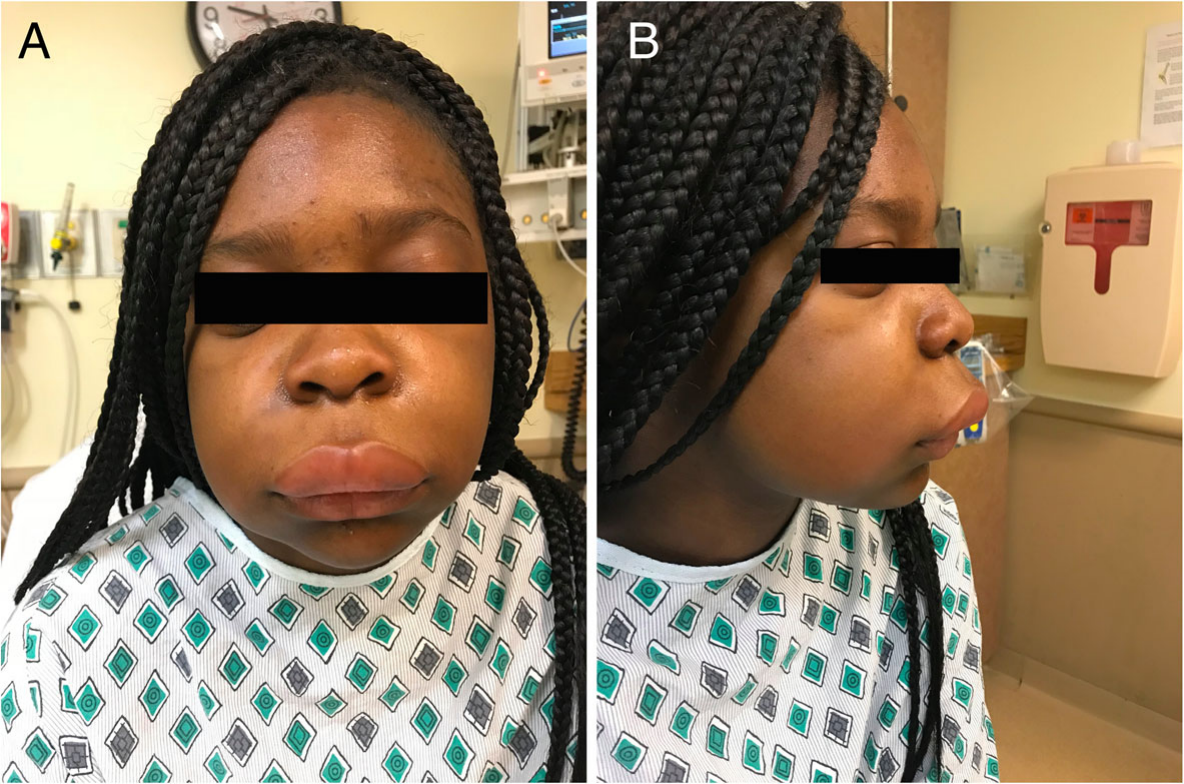
A 14-year-old female with spontaneous lip swelling (upper > lower) extending to the philtrum and premaxilla on frontal (**A**) and lateral (**B**) views

A 14-year-old African American female presented to the emergency department with spontaneous, sudden-onset lip swelling (upper greater than lower lip) for 1 h. On examination, there was significant water-bag edema of the upper lip extending to the philtrum and premaxilla (Fig. 1). The patient and her mother indicated that she did not have any food allergies nor did she have any new or unusual exposures. There was no family history of angioedema. Nasopharyngeal laryngoscopy revealed a patent airway without edema. She was initiated on intravenous dexamethasone, famotidine, and diphenhydramine which improved her edema but did not completely resolve it. She was subsequently transferred to a local pediatric hospital and upon further testing she was found to have a C1 esterase inhibitor de novo gene mutation. She was scheduled to see an allergist as an outpatient for further management.

## Diagnosis

The prevalence of hereditary angioedema is very low, affecting 1/10,000-50,000 people. Hereditary angioedema causes localized, non-pitting edema of the dermis, subcutaneous and submucosal tissue, and often manifests in the lips, face, mouth, and throat. Two main pathways contribute to angioedema—the most immediate pathway (minutes to hours) is mediated by mast cell and basophil degranulation causing urticaria and pruritus, while the more delayed pathway (hours-days) is mediated by kinin. Hereditary angioedema may be considered in the case of angioedema without urticaria, as this rare form involves the latter pathway. Unlike the acquired type, hereditary angioedema is bradykinin-mediated only and is mostly caused by mutations to C1 inhibitor (C1INH), which, through the complement pathway, leads to low C4 levels and eventually uninhibited bradykinin. Hereditary angioedema may be diagnosed by measuring C4 and C1INH levels [1].

Hereditary angioedema typically presents before adulthood as recurrent angioedema attacks without pruritus or urticaria. These episodes usually last 2-5 days without treatment, with no associated triggers and with a possible family history of angioedema. Initial management requires assessing the airway, because edema may rapidly progress. Airway obstruction occurs in up to 15% of cases, accounting for a large portion of morbidity and mortality [2].

Signs of laryngeal involvement include change in voice, stridor, dysphagia, and dyspnea. In these cases, fiberoptic visualization should be emergently performed and may reveal pharyngeal or more ominously, laryngeal edema involving the vocal folds, epiglottis, aryepiglottic folds, or arytenoids. When laryngeal edema is present it may necessitate definitive airway management [3]. If suspecting a need for intubation, laryngoscopy may be performed with endotracheal tube loaded onto it to facilitate fiberoptic intubation. Patients should be monitored in the intensive care unit. Other treatments include C1 inhibitors, bradykinin B_2_ receptor antagonist, and kallikrein inhibitors to block the bradykinin activity. If these are unavailable, fresh frozen plasma may be used to replace C1 inhibitor [4, 5]. Unlike histamine-mediated angioedema, the hereditary form typically does not respond to antihistamine, corticosteroids, and epinephrine.

## Data Availability

Not applicable
